# Life-history trait plasticity and its relationships with plant adaptation and insect fitness: a case study on the aphid *Sitobion avenae*

**DOI:** 10.1038/srep29974

**Published:** 2016-07-18

**Authors:** Peng Dai, Xiaoqin Shi, Deguang Liu, Zhaohong Ge, Da Wang, Xinjia Dai, Zhihao Yi, Xiuxiang Meng

**Affiliations:** 1 State Key Laboratory of Crop Stress Biology for Arid Areas (Northwest A&F University), Yangling, Shaanxi 712100, China; 2 College of Plant Protection, Northwest A&F University, Yangling, Shaanxi 712100, China; 3 Department of Foreign Languages, Northwest A&F University, Yangling, Shaanxi Province 712100, China; 4 School of Environment and Natural Resources, Renmin University of China, Beijing 100872, China

## Abstract

Phenotypic plasticity has recently been considered a powerful means of adaptation, but its relationships with corresponding life-history characters and plant specialization levels of insects have been controversial. To address the issues, *Sitobion avenae* clones from three plants in two areas were compared. Varying amounts of life-history trait plasticity were found among *S. avenae* clones on barley, oat and wheat. In most cases, developmental durations and their corresponding plasticities were found to be independent, and fecundities and their plasticities were correlated characters instead. The developmental time of first instar nymphs for oat and wheat clones, but not for barley clones, was found to be independent from its plasticity, showing environment-specific effects. All correlations between environments were found to be positive, which could contribute to low plasticity in *S. avenae*. Negative correlations between trait plasticities and fitness of test clones suggest that lower plasticity could have higher adaptive value. Correlations between plasticity and specialization indices were identified for all clones, suggesting that plasticity might evolve as a by-product of adaptation to certain environments. The divergence patterns of life-history plasticities in *S. avenae*, as well as the relationships among plasticity, specialization and fitness, could have significant implications for evolutionary ecology of this aphid.

One of the fundamental objectives in studies of evolutionary ecology is to determine the causes and implications of phenotypic changes among natural populations^1^. Populations of different organisms may experience variable natural environments in space and time, where they often respond with adaptive phenotypic divergence as a result of complex interactions between their genomes and the environment^2^,^3^. The process can involve both local adaptation (i.e., genetic differentiation) and adaptive phenotypic plasticity^1^,^3^. This is because phenotypic plasticity (broadly defined as all phenotypic responses to environmental changes) may facilitate the successful establishment of a species in unpredictable, heterogeneous or novel environments^3^,^4^,^5^. Phenotypic plasticity has been recently considered as a powerful means of adaptation for different organisms (especially for plants and insects) in various environments, despite that its functional roles have long been considered non-significant in the evolutionary ecology^5^,^6^,^7^. Indeed, as a large group of insects, aphids can show phenotypic plasticity in many aspects of their lives. For example, a significant amount of plasticity was found in insecticide susceptibility for genetically identical cotton aphids (*Aphis gossypii* Glover)^8^. *Aphis fabae* genotypes were highly plastic in host choice behavior^7^. Clones of the pea aphid, *Acyrthosiphon pisum* (Harris), showed natural enemy induced phenotypic plasticity by producing a greater proportion of alate offspring, when responding to the chemical traces present in tracks left by ladybird beetles^9^. Local populations of the cotton aphid (*A. gossypii*) in Australia were found to show environmentally induced changes in morphology^10^. Clones of *A. fabae* demonstrated high levels of phenotypic plasticity in some life-history traits such as the intrinsic rate of natural increase and developmental time^6^. Therefore, aphids’ success in a wide variety of agricultural ecosystems can be at least partially attributed to their broad phenotypic plasticity in morphological, physiological, behavioral or other life-history characters.

Although they are highly plastic in various characters, many aphid species are relatively specialized to certain plants^2^,^11^. This phenomenon seems to be in agreement with the specialization hypothesis where a relatively specialized genotype for a certain environment should have relatively high plasticity across a range of alternative environments, particularly for life-history traits that are closely related to the genotype’s fitness^12^. This hypothesis is in agreement with studies of Nylin^13^ and West-Eberhard^14^ where host plant specialization (ultimately speciation) can be driven by phenotypic plasticity in host utilization. Nonetheless, this idea is still controversial since plasticity may dampen natural selective effects by allowing individuals to rapidly adapt to novel environments, thereby constraining adaptive genetic changes^15^,^16^. In addition, general patterns of phenotypic plasticity in natural populations along environmental gradients still remain elusive despite remarkable expansion of plasticity research in recent years^17^.

*Sitobion avenae* (Fabricius), a widespread pest aphid on cereals (e.g., wheat, oat and barley) around the world^11^,^18^,^19^,^20^, is a good model to address these issues. This is because this aphid can survive on a lot of wild plants in the Poaceae, and specialize to a certain degree on all cereal crops^11^,^21^, which constitute heterogeneous (and often discrete) environments where *S. avenae* may have to respond with phenotypic plasticity. In our previous study^3^, we collected *S. avenae* clones on wheat, barley and oat from two provinces of China, tested them in common laboratory conditions, and analyzed the genetic basis and selection for plasticity of *S. avenae*’s life-history traits on the three plants. In this study, we focus on the amount and patterns of *S. avenae*’s life-history trait plasticity, as well as the relationships among phenotypic plasticity, plant specialization and relative fitness of *S. avenae* clones. Specifically, the aims of this study were to: (i) determine the amounts of phenotypic plasticity of *S. avenae*’s life-history traits on alternative host plants; (ii) explore the patterns of plasticity of different *S. avenae* clones on the three plants; (iii) examine the relationships among phenotypic plasticity, specialization and fitness of *S. avenae*.

## Results

### Comparisons in the amount of plasticity

The phenotypic plasticity of life-history traits [i.e., the developmental duration of 1^st^ to 4^th^ instar nymphs (DT1-DT4), the total developmental duration of nymphs (DT5), and 7 d fecundity] of test *S. avenae* clones was analyzed. Significant differences in plasticity levels of test life-history traits were found among *S. avenae* clones (i.e., barley, oat and wheat), and between both areas (i.e., Qinghai and Shaanxi) as well (Table 1). For *S. avenae* clones collected from the Qinghai area, barley clones showed higher plasticity in DT1 (*F* = 5.97; df = 2, 330; *P* < 0.01), but lower plasticity in DT2 (*F* = 3.80; df = 2, 330; *P* < 0.05), compared to wheat (or oat) clones. Oat clones had lower plasticity in DT4 (*F* = 14.84; df = 2, 330; *P* < 0.001) and DT5 (*F* = 12.42; df = 2, 330; *P* < 0.001) than wheat clones. No significant differences in plasticity of DT3 or 7-d fecundity were found among barley, oat or wheat clones from Qinghai. Barley clones from Shaanxi had significantly higher plasticity in DT3 (*F* = 3.31; df = 2, 330; *P* < 0.05), DT4 and 7-d fecundity (*F* = 46.48; df = 2, 330; *P* < 0.001), but not in DT1, DT2, or DT5, compared to wheat clones from the same area. Oat clones from Shaanxi were also found to be less plastic in DT4 and 7-d fecundity than barley clones of the same area. *Sitobion avenae* clones from Qinghai tended to be more plastic in all tested life-history traits but DT4 and 7-d fecundity, compared to those from Shaanxi.

**Figure 1:**
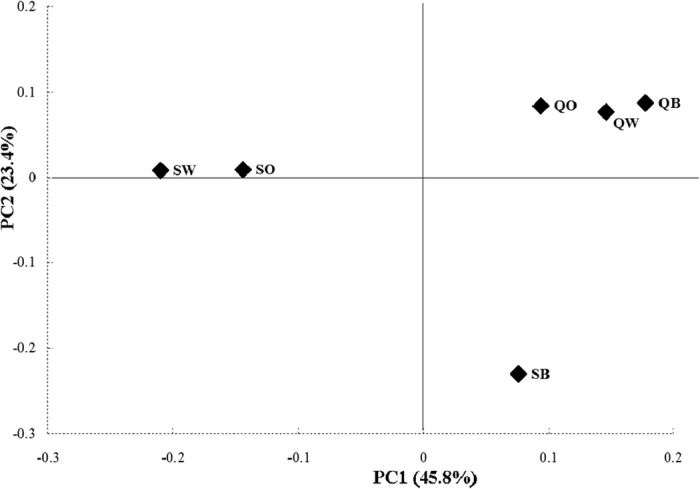
Plot of PC1 vs PC2 from principal component analysis of life-history trait plasticities for all *Sitobion avenae* clones combined (PC1, PC2 and PC3 explained 45.8%, 23.4%, and 14.0% of the total variation, respectively; QB, barley clones of Qinghai; QO, oat clones of Qinghai; QW, wheat clones of Qinghai; SB, barley clones of Shaanxi; SO, oat clones of Shaanxi; SW, wheat clones of Shaanxi).

The first three principal components (PC1 to PC3) explained 83.2% of the total variation in life-history trait plasticities of *S. avenae* clones collected from three plants in two areas (Fig. 1). The plasticities of DT1, DT3, DT4 and 7-d fecundity contributed the most to PC1 with positive correlations. The second principal component (PC2) was associated mainly with plasticities of DT1 (positive) and 7-d fecundity (negative). The plot of PC1 vs. PC2 showed that barley, oat and wheat clones from the Qinghai area clustered together in the upper right of the plot, indicating little variation in life-history trait plasticities of these clones. Barley clones of the Shaanxi area fell in the lower right of the plot, whereas wheat and oat clones from Shaanxi clustered together near the middle left of the plot.

### Environmental correlations comparing patterns of plasticity

Significant correlations between the measurements on the original plant and those on the alternative plants were found for DT4, DT5, and 7-d fecundity, but not for DT1, DT2 or DT3 (Table 2). Positive correlations of DT4 were found for oat clones from Qinghai (*r* = 0.3704; *P* < 0.05), but not for all other clones. Correlations of DT5 were significantly positive for oat clones from Qinghai (*r* = 0.5095; *P* < 0.01), and barley clones from Shaanxi (*r* = 0.3581; *P* < 0.05). All clones from both areas showed positive correlations of 7 d fecundity (*r* = 0.3889 to 0.8213; *P* < 0.05) except barley and oat clones from Shaanxi.

### Associations between life-history traits and their plasticity

DT1 was found to be correlated with its plasticity for barley clones (Table 3; Spearman correlation: ρ = −0.6211, *P* < 0.05; Hoeffding test: *D* = 0.1259, *P* < 0.05), but not for oat or wheat clones. The dependence of DT3 on its plasticity (and vice versa) was found for oat clones (Hoeffding test: *D* = 0.0885, *P* < 0.05), although the Spearman correlation between both characters was non-significant. In all the other cases, the developmental durations and their corresponding plasticities were found to be independent characters.

**Figure 2:**
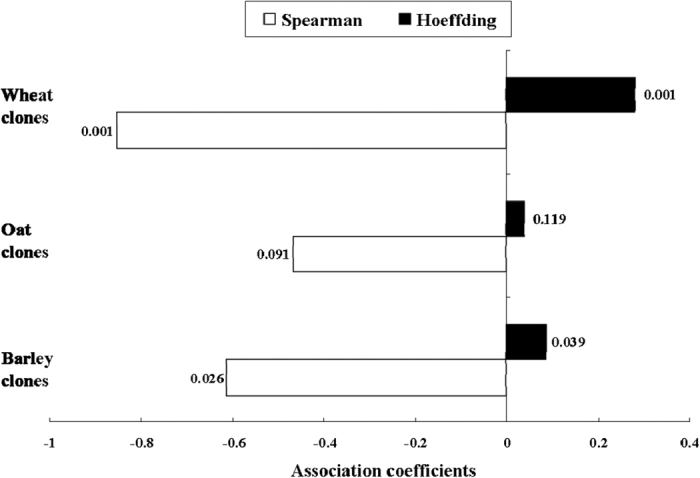
Associations between fecundity and its plasticity for *Sitobion avenae* clones collected from barley, oat and wheat (Spearman correlation analyses and Hoeffding tests of independence were conducted in SAS at α = 0.05).

Significant correlations between fecundities and their corresponding plasticities were found for *S. avenae* clones from barley (Fig. 2; Spearman correlation: ρ = −0.6143, *P* < 0.05; Hoeffding test: *D* = 0.0871, *P* < 0.05) and wheat (Spearman correlation: ρ = −0.8536, *P* < 0.001; Hoeffding test: *D* = 0.2822, *P* < 0.001). Such associations were not significant for oat clones, indicating the independence of both characters in this case.

### Relationships among plasticity, specialization and fitness of *S. avenae* clones

All significant correlations between the developmental time (DT1 to DT5) plasticity and relative fitness were negative for *S. avenae* clones from three plants in two areas (Table 4), showing a significant cost of plasticity in *S. avenae*. For barley clones from the Shaanxi area, the relative fitness of *S. avenae* was found to be significantly correlated with the plasticity of all developmental durations (*r* = −0.5167 to −0.6494, *P* < 0.001) but DT1 and DT2, whereas none of the correlations were significant for oat clones in the same area. The relative fitness of wheat clones from the Shaanxi area was significantly correlated with DT4 plasticity (*r* = −0.4062, *P* < 0.001). The relative fitness of barley clones from the Qinghai area was significantly correlated with the plasticity of DT1 (*r* = −0.5445, *P* < 0.001), DT4 (*r* = −0.5227, *P* < 0.001) and DT5 (*r* = −0.4216, *P* < 0.01). For oat clones from Qinghai, correlations with relative fitness were significant plastcities of DT4 (*r* = −0.6444, *P* < 0.001) and DT5 (*r* = −0.4076, *P* < 0.01). The relative fitness of wheat clones from Qinghai was significantly correlated to the plasticity of DT2 (*r* = −0.2961, *P* < 0.05) and DT5 (*r* = −0.5451, *P* < 0.001). Significantly negative correlations between relative fitness and PC1 (*r* = −0.2750 to −0.6067, *P* < 0.05) were found for all test clones but those from oat of Shaanxi and wheat of Qinghai. The only significant correlation between PC2 and relative fitness was identified for barley clones from Shaanxi (*r* = −0.5844, *P* < 0.001). The correlation between PC3 and relative fitness was negative for barley clones from Shaanxi (*r* = −0.5572, *P* < 0.001), but it was positive for oat (*r* = 0.2759, *P* < 0.05) and wheat (*r* = 0.2611, *P* < 0.05) clones of shaanxi, and barley clones of Qinghai (*r* = 0.5251, *P* < 0.001).

For barley clones of *S. avenae*, the correlations with the specialization index (Xsp) were significantly positive for DT2 plasticity (Table 5; *r* = 0.5812, *P* < 0.05), and PC3 (*r* = 0.5744, *P* < 0.05). Significantly negative correlations for oat clones were identified between Xsp and plasticity of DT4 (*r* = −0.5545, *P* < 0.05), as well as between Xsp and PC2 (*r* = −0.5345, *P* < 0.05). Xsp of wheat clones was negatively correlated to plasticities of DT4 (*r* = −0.5754, *P* < 0.05) and DT5 (*r* = −0.6406, *P* < 0.05).

**Figure 3:**
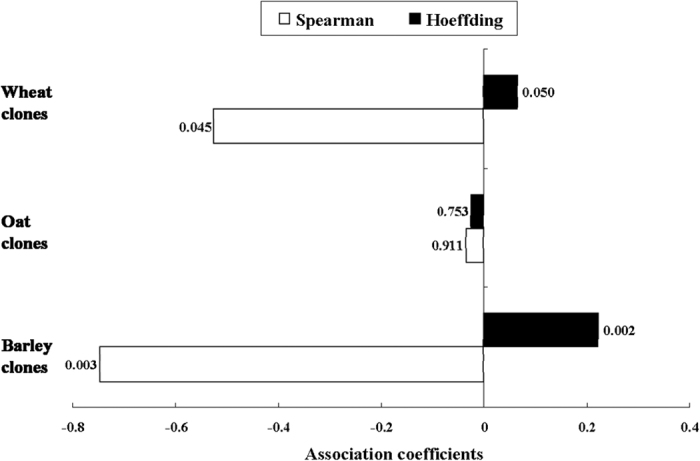
The relationship between the plasticity of fecundity and host plant specialization (Xsp) of *Sitobion avenae* clones collected from barley, oat and wheat (Spearman correlation analyses and Hoeffding tests of independence were conducted in SAS at α = 0.05).

The plasticity of fecundity was found to be significantly correlated with the extent of specialization (Xsp) for both barley (Fig. 3; Spearman correlation: ρ = −0.7466, *P* < 0.01; Hoeffding test: *D* = 0.2228, *P* < 0.01) and wheat (Spearman correlation: ρ = −0.5250, *P* < 0.05; Hoeffding test: *D* = 0.0654, *P* = 0.05) clones of *S. avenae*, meaning that higher fecundity plasticities should be associated with lower Xsp in these clones. However, the correlations were not significant for oat clones, and this indicated the independence between fecundity and its plasticity for these *S. avenae* clones.

**Figure 4:**
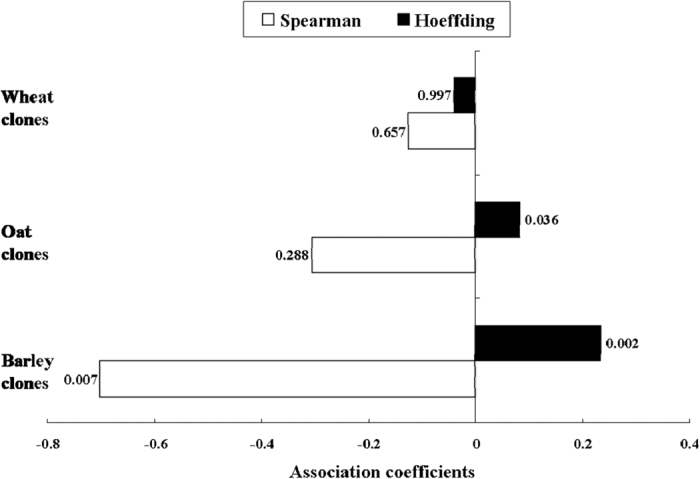
The relationship between the plasticity of fecundity and relative fitness of *Sitobion avenae* clones collected from barley, oat and wheat (Spearman correlation analyses and Hoeffding tests of independence were conducted in SAS at α = 0.05).

The plasticity of fecundity was found to be significantly correlated to the relative fitness for barley clones (Fig. 4; Spearman correlation: ρ = −0.7025, *P* < 0.01; Hoeffding test: *D* = 0.2341, *P* < 0.01), suggesting a cost of plasticity for these clones. Although the Spearman correlations (ρ = −0.3055, *P* = 0.288) between both characters were not significant, fecundity plasticity and relative fitness were not independent of each other for oat clones based on Hoeffding test (*D* = 0.0834, *P* < 0.05). The fecundity plasticity was found to be independent of relative fitness for wheat clones.

## Discussion

Under heterogeneous environmental conditions, aphids are prone to be plastic in their various life-history characters^2^,^3^,^7^. In this study, *S. avenae* was shown to be more or less plastic in all test life-history traits. Among these traits, relatively high amount of plasticity could occur in DT1 and fecundity for certain clones (Table 1). Varying amounts of life-history trait plasticity were found among barley, oat and wheat clones. For example, barley clones from Shaanxi showed significantly higher plasticity in DT4 and fecundity than oat or wheat clones. Wheat clones from Qinghai were found to be more plastic in DT4 and DT5 than oat clones from the same area. Additional evidence for the differentiation of *S. avenae* clones was the clustering patterns for *S. avenae* clones in the PCA plot, where separation of barley clones of Shaanxi from wheat or oat clones of the same area was evident in the plot. The identified differences among *S. avenae* clones indicated that they had differentiated to a certain degree in terms of phenotypic plasticity under the selection of the three test plants (for more details about the selective effects, see^3^). These results were consistent with the findings that the divergence of populations from various host plants was evident for *S. avenae*^11^,^19^.

Interestingly, plasticity of *S. avenae* clones also showed geographic differences in our study. For example, barley clones were found to be more plastic in fecundity than oat or wheat clones from Shaanxi, but no such differences were found among these clones from Qinghai. Geographic differentiation in plasticity of *S. avenae* clones was also demonstrated by the clear separation of Qinghai clones from Shaanxi clones in the PCA plot. One explanation is that *S. avenae* might have adapted to local environmental conditions in different geographic regions, and the resulting ecotypes might have evolved distinct patterns of life-history plasticity. This is likely because local adaptation appears to be common for this aphid^22^,^23^,^24^,^25^. Another mutually non-exclusive explanation is that geographic differences could occur between both areas in the composition of secondary endosymbionts for *S. avenae*, because certain symbionts (e.g., *Regiella insecticola*) have been demonstrated to influence life-history plasticity of their host aphids^26^.

In our study, *S. avenae* clones showed also divergence in associations between life-history traits and their plasticity. For example, barley clones, but not oat or wheat clones, presented significant correlations between DT1 and its plasticity. Fecundities were found to correlate with their plasticities for barley and wheat clones, but not for oat clones. In a majority of the cases, developmental durations and their corresponding plasticities were found to be independent characters, but fecundities and their corresponding plasticities were correlated characters. Both models of selection on character states and models of selection on coefficients of reaction norms have been developed in studies on phenotypic plasticity. Thus, it has been controversial to consider plasticities of life-history traits as separate characters themselves^27^,^28^,^29^. Our study showed that the independence of plasticities from their corresponding characters was environment (i.e., host plant) specific, and as well as life-history trait specific.

Phenotypic plasticity has been considered as a significant means of adaptation for different organisms in novel environments, and it may be a major determinant of the evolutionary trajectory for the species involved^3^,^6^. However, plasticity of fitness traits in *S. avenae* appeared to be low in our study. The optimal level of plasticity is thought to be a compromise between the environmental sensitivity of phenotypic selection and the correlation between original and alternative environments^30^. After summer harvests of cereal crops, *S. avenae* individuals need to disperse short or long distances to find alternative host plants such as wild grasses or other cereals. These individuals may have to feed on whatever host plants they can find in order to survive under such seasonal and ephemeral conditions. The resulting low predictability of the environment could lead to the occurrence of low plasticity in *S. avenae*, and the reason is that if environmental predictability is poor, it can be detrimental to be very responsive to the environment^30^.

Particular positive correlations between environments (Table 2) were identified for all *S. avenae* clones from both areas except those of oat in Shaanxi, providing further evidence of differentiation among test clones. This could also account for low plasticity of *S. avenae*, because genetic correlations among environments can constrain the evolution of high levels of plasticity^13^. Despite negative impacts of environmental correlations on plasticity in this aphid, the divergence in plasticity among *S. avenae* clones could be the key for population persistence in changing and often unpredictable environments experienced by this aphid^3^,^24^. It is believed that ability of many species to survive in fluctuating environments will be closely related to patterns of plasticity for fitness traits such as developmental time and fecundity^31^. It is challenging to elucidate the general patterns of plasticity for populations of organisms in nature, but such patterns of phenotypic flexibility could be of significance to develop ecological and evolutionary models for predicting the abundance and distribution of the organisms involved^31^.

In our study, plasticities of particular developmental durations were found to be significantly correlated to fitness for all test *S. avenae* clones of both areas except oat clones from Shaanxi. The plasticity of fecundity was negatively correlated to fitness for barley clones only. All significant correlations between plasticity and fitness were negative, suggesting that the cost of maintaining plasticity in *S. avenae* could be high. This is in contrast to a study on another aphid, *Brevicoryne brassicae* L., where no significant relationships between trait plasticity and fitness were identified (meaning no cost of plasticity)^32^. Our results also indicate that higher levels of phenotypic plasticity for *S. avenae* clones could have lower adaptive value. Despite the low adaptive value of high plasticity, our results can not exclude the possibility that phenotypic plasticity might initially have an important role during *S. avenae*’s colonization of a new plant during the evolutionary process, since the fitness of test *S. avenae* clones on alternative plants were quite high^3^. Interestingly, significantly negative correlations between the plasticities of developmental durations (e.g., DT4 or DT5) and specialization indices (Xsp) were found for oat and wheat clones, and the same relationship between the plasticity of fecundity and Xsp occurred for barley and wheat clones. The close relationships between life-history trait plasticity and Xsp identified in our study support the idea that phenotypic plasticity might evolve as a by-product of adaptation to certain environments^1^. However, our results are not in agreement with the specialization hypothesis by Taylor and Aarssen^12^, where specialized genotypes are expected to have high levels of plasticity. On the contrary, *S. avenae* clones with higher extents of specialization tended to have lower plasticity in fitness traits in our case. This study supports the hypothesis by Lortie and Aarssen^16^ that the evolution of relatively specialized genotypes can be accompanied by decrease or no change in life-history trait plasticity.

However, adaptive plasticity is often interpreted for fitness parameters like fecundity^16^, and *S. avenae* has been shown to have evolutionary potential of adaptive plasticity^3^. Despite negative relationships between plasticity and fitness, relationships between plasticity of particular life-history traits and Xsp were significant for all test *S. avenae* clones. Thus, higher phenotypic plasticity in certain *S. avenae* clones may not indicate greater adaptation, but it may rather indicate lower level of specialization. Overall, the relationships among phenotypic plasticity, and host plant specialization levels and fitness of *S. avenae* clones appeared to be closely linked, and might evolve closely together. Genetic bases for life-history trait plasticity in *S. avenae* and selective effects of different host plants have been demonstrated in^3^, and further studies along molecular fronts should be conducted to determine mechanisms underlying the patterns of plasticity in *S. avenae* on various host plants. Although the ecological roles and evolution of phenotypic plasticity are still controversial currently^33^, our results indicate that complex interactions may occur among plasticity, specialization and fitness parameters, which can have significant implications for the ecology and evolution of various organisms. Our results will be of significance in constructing more sophisticated models of plasticity and phenotypic evolution, which take into account the associations between plasticities and their corresponding life-history characters, natural patterns of plasticity, and environmental specific effects.

## Methods

### Aphid and plant

Clonal genotypes of *S. avenae* were collected from wheat, oat and barley fields in provinces of Shaanxi and Qinghai from May to August of 2013, and separate colonies of single clones were established in the laboratory as detailed in^3^. Briefly, at least 20 different clones from each plant were collected in each area. Plants of barley (*Hordeum vulgare* L. cv. Xian 91-2), wheat (*Triticum aestivum* cv. Aikang 58), and oat (*Avena sativa* L. cv. Sandle) were cultured in plastic pots (6 cm in diameter) filled with turfy soil, vermiculite and perlite (4:3:1, v/v/v). Individual aphid colonies were maintained on the source plant species (i.e. wheat, oat or barley) in rearing rooms (at 20 ± 2 °C and under a 16:8 light: dark cycle). Each aphid colony was covered with a transparent plastic cylinder (15 cm in height, 5.5 cm in diameter), to which a Terylene mesh top was glued for ventilation. In order to minimize the confounding effects of varying environmental factors among sampling sites, aphid clones were reared for at least two generations in common laboratory conditions before the following life-history bioassays^11^,^34^.

### Life history bioassays

Life-history bioassays were conducted as detailed previously in^3^. Briefly, 16 different clones (10 from Shaanxi and 6 from Qinghai) for each plant species were randomly selected for use in the bioassays. When they were at one- or two-leaf stage, single plant seedlings of barley, oat or wheat received one new-born first instar nymph each. Each pot of plants with aphids on them was enclosed with a plastic cylinder. Tests were conducted in environmental growth chambers (BIC 400, Shanghai Boxun Medical Biological Instrument Corp.) under the following conditions: 20 ± 1 °C, a light: dark cycle of 16:8 (h), and the relative humidity of 65% (±2%). The test of each *S. avenae* clone was repeated four to six times on each plant. Each test individual was monitored daily until its death, molting and mortality events were recorded, and all produced offspring were counted and then removed.

### Statistical analysis

The duration of development (in days) for first to fourth instar nymphs (hereafter referred to as DT1 to DT4), the total developmental duration for the nymphal stage (hereafter referred to as DT5), and 7-d fecundity (offspring accumulated in 7 days since the initiation of reproduction) were tabulated. The amount of plasticity of each *S. avenae* clone was determined by analyzing the coefficient of variation (CV) for the abovementioned traits in alternative environments (i.e., on different test plants) as described previously in^3^. Two way nested analysis (clones nested in plant origin) of variance (nested ANOVA) was used to analyze the amount of plasticity with SAS^35^. The main effects of plant origin and sampling location were determined in ANOVA where the requirements of normality and homoscedasticity for data were satisfied. The plasticity means were compared with Tukey’s honestly significant difference (HSD) test at α = 0.05 after a significant ANOVA.

In order to explore the clustering patterns of test *S. avenae* clones, the Proc PRINCOMP procedure in SAS was used to conduct principal component analyses (PCA) with all test life-history plasticities of barley, oat and wheat clones from both areas. The pattern of plastic responses (i.e., another measure of phenotypic plasticity) to alternative plants by *S. avenae* clones was determined with correlation analyses following Schlichting and Levin^36^. This measure can complement the abovementioned evaluation of plasticity, and avoid interpretation problems that might arise from the utilization of CV alone^36^. The associations between *S. avenae*’s life-history traits (i.e., nymphal developmental durations and fecundity) and their corresponding plasticity were determined using both Spearman correlation analyses and Hoeffding tests of independence^35^.

Relative fitness of a *S. avenae* clone was determined using the clone’s 7 d fecundity as described in^3^. The relationships between developmental duration plasticity and relative fitness of *S. avenae* clones were identified using Pearson correlation analyses. Another PCA analysis with DT1 to DT5 plasticities was also conducted, and composite plasticity factors (i.e., the first three principal components) were extracted and then used in the abovementioned correlation analyses.

As described previously in^11^, 7-d fecundity was considered as the fitness surrogate in evaluating the extent of specialization (Xsp) for *S. avenae* clones. Xsp values of oat clones were calculated as follows (modified from^11^,^37^):
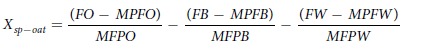
X_sp-oat_, Xsp of oat clones; FO, fitness on oat; MPFO, mean population fitness on oat; MFPO, mean fitness of population oat; FB, fitness on barley; MPFB, mean population fitness on barley; MFPB, mean fitness of population barley; FW, fitness on wheat; MPFW, mean population fitness on wheat; MFPW, mean fitness of population wheat.

Similarly, Xsp values of barley and wheat clones were determined. Relatively specialized clones will have higher values of Xsp than those relatively generalized clones. Pearson correlation analyses were utilized to assess the relationships between Xsp and developmental duration plasticity of *S. avenae* clones. The associations among Xsp, plasticity of 7 d fecundity, and relative fitness of *S. avenae* clones were analyzed using two non-parametric tests of independence (i.e., Spearman correlations and Hoeffding’s *D* statistics)^35^. The Pearson and Spearman correlation coefficients, as well as the *D* statistic of Hoeffding tests, were calculated using the PROC CORR procedure in SAS^35^.

## Additional Information

**How to cite this article**: Dai, P. *et al*. Life-history trait plasticity and its relationships with plant adaptation and insect fitness: a case study on the aphid *Sitobion avenae*. *Sci. Rep.* **6**, 29974; doi: 10.1038/srep29974 (2016).

## Figures and Tables

**Table 1:** Average amount of plasticity for developmental durations of nymphs and fecundity of *Sitobion avenae* clones collected from barley, oat and wheat in Qinghai and Shaanxi areas (Data entries are coefficients of variation; DT1-DT4, the developmental duration of 1^st^ to 4^th^ instar nymphs; DT5, the total developmental duration of nymphs; data with different letters within a column were significantly different at α = 0.05, ANOVA followed by Tukey tests).

**Source**	**Clones**	**DT1**	**DT2**	**DT3**	**DT4**	**DT5**	**7 d-fecundity**
Qinghai	barley	0.416 a	0.204 b	0.311 a	0.272 ab	0.126 b	0.324 b
	oat	0.298 b	0.282 a	0.320 a	0.210 bc	0.118 b	0.293 b
	wheat	0.297 b	0.293 a	0.325 a	0.294 a	0.173 a	0.294 b
Shaanxi	barley	0.172 c	0.129 c	0.182 b	0.291 a	0.090 bc	0.502 a
	oat	0.182 c	0.139 c	0.129 bc	0.201 c	0.103 bc	0.184 c
	wheat	0.126 c	0.162 bc	0.120 c	0.155 c	0.071 c	0.161 c

**Table 2:** Correlations between character means of *Sitobion avenae* clones on the original plant and those on alternative plants comparing patterns of their life-history trait plasticity (Pearson product-moment correlation coefficients were calculated using life-history trait values; DT1-DT4, the developmental duration of 1^st^ to 4^th^ instar nymphs; DT5, the total developmental duration of nymphs; *P < 0.05; **P < 0.01).

**Source**	**Clones**	**DT1**	**DT2**	**DT3**	**DT4**	**DT5**	**7-d fecundity**
Qinghai	Barley	0.0992	−0.0261	0.1030	−0.0666	0.0673	0.5042*
	Oat	−0.1451	0.2394	0.1686	0.3704*	0.5095**	0.6953**
	Wheat	0.0682	−0.1065	0.0098	−0.0450	0.0938	0.8213**
Shaanxi	Barley	0.1102	−0.2893	−0.1205	−0.1661	0.3581*	0.2165
	Oat	0.1197	0.1600	0.0214	0.2055	0.1014	−0.1812
	Wheat	−0.0121	−0.2579	0.1318	0.0703	0.1802	0.3889**

**Table 3:** Pearson correlation coefficients (*P* values) between the developmental time plasticity and Xsp (the specialization index) for *Sitobion avenae* clones from three plants [DT1-DT4, the developmental duration of 1^st^ to 4^th^ instar nymphs; DT5, the total developmental duration of nymphs; principal component analyses were conducted using plasticities of DT1 to DT5; the first three principal components (PC) explained 86.6% of the total variation; significant correlations are highlighted in boldface].

**Traits**	**Barley clones**	**Oat clones**	**Wheat clones**
DT1	0.5278 (0.064)	0.4679 (0.092)	−0.2488 (0.371)
DT2	**0.5812 (0.037)**	0.4512 (0.105)	−0.4874 (0.065)
DT3	0.1106 (0.719)	0.4624 (0.096)	−0.4188 (0.120)
DT4	−0.3699 (0.213)	**−0.5545 (0.040)**	**−0.5754 (0.025)**
DT5	−0.1684 (0.582)	−0.2537 (0.381)	**−0.6406 (0.010)**
PC1	0.2414 (0.427)	0.3816 (0.178)	−0.5392 (0.070)
PC2	−0.5134 (0.073)	**−0.5345 (0.049)**	−0.2912 (0.292)
PC3	**0.5744 (0.040)**	0.4968 (0.071)	−0.3268 (0.234)

**Table 4:** Pearson correlations between the developmental time plasticity and relative fitness of *Sitobion avenae* clones from three host plants in two areas (DT1-DT4, the developmental duration of 1^st^ to 4^th^ instar nymphs; DT5, the total developmental duration of nymphs; PC1 to PC3, first to third factor extracted from principal component analysis of DT1 to DT5 plasticities; *P < 0.05; **P < 0.01; ***P < 0.001).

**Traits**	**Clone sources**					
	**Shaanxi area**			**Qinghai area**		
	**Barley**	**Oat**	**Wheat**	**Barley**	**Oat**	**Wheat**
DT1	−0.2252	−0.2427	−0.2149	−0.5445***	−0.2120	0.0164
DT2	0.1729	0.1915	−0.2113	0.1893	−0.0932	−0.2961*
DT3	−0.5807***	0.2102	0.0615	0.0346	−0.1061	0.0082
DT4	−0.6494***	−0.0364	−0.4062***	−0.5227***	−0.6444***	−0.1955
DT5	−0.5167***	−0.0766	−0.1782	−0.4216**	−0.4076**	−0.5451***
PC1	−0.6067***	−0.0358	−0.2750*	−0.3629*	−0.3931**	−0.1570
PC2	−0.5844***	0.1819	−0.0481	0.2297	−0.0830	−0.1312
PC3	−0.5572***	0.2759*	0.2611*	0.5251***	0.2760	−0.1880

**Table 5:** Association coefficients between the developmental time and its plasticity for *Sitobion avenae* clones from three plants (Spearman correlation analyses and Hoeffding tests of independence conducted in SAS; DT1-DT4, the developmental duration of 1^st^ to 4^th^ instar nymphs; DT5, the total developmental duration of nymphs; significant correlations highlighted in boldface; *P < 0.05).

**Traits**	**Barley clones**		**Oat clones**		**Wheat clones**	
	**Spearman**	**Hoeffding**	**Spearman**	**Hoeffding**	**Spearman**	**Hoeffding**
DT1	−0.6211*	0.1259*	−0.3274	0.0308	−0.2634	0.0186
DT2	0.1728	−0.0069	−0.3992	−0.0292	0.3135	−0.0087
DT3	0.2545	−0.0526	−0.3920	0.0885*	−0.3910	0.0364
DT4	0.0222	−0.0349	0.0425	−0.0335	−0.2542	−0.0538
DT5	0.1685	0.0720	0.3815	−0.0235	−0.2164	−0.0073
